# Online daily assessment of dose change in head and neck radiotherapy without dose‐recalculation

**DOI:** 10.1002/acm2.12432

**Published:** 2018-08-07

**Authors:** Jason R. Vickress, Jerry Battista, Rob Barnett, Slav Yartsev

**Affiliations:** ^1^ Department of Medical Biophysics Western University London ON Canada; ^2^ Department of Oncology Western University London ON Canada; ^3^ London Regional Cancer Program London Health Sciences Centre London ON Canada

**Keywords:** adaptive radiotherapy, cone beam CT, deformable image registration, head and neck cancer, image‐guided radiotherapy, radiotherapy

## Abstract

**Background:**

Head and neck cancers are commonly treated with radiation therapy, but due to possible volume changes, plan adaptation may be required during the course of treatment. Currently, plan adaptations consume significant clinical resources. Existing methods to evaluate the need for plan adaptation requires deformable image registration (DIR) to a new CT simulation or daily cone beam CT (CBCT) images and the recalculation of the dose distribution. In this study, we explore a tool to assist the decision for plan adaptation using a CBCT without re‐computation of dose, allowing for rapid online assessment.

**Methods:**

This study involved 18 head and neck cancer patients treated with CBCT image guidance who had their treatment plan modified based on a new CT simulation (ReCT). Dose changes were estimated using different methods and compared to the current gold standard of using DIR between the planning CT scan (PCT) and ReCT with recomputed dose. The first and second methods used DIR between the PCT and daily CBCT with the planned dose or recalculated dose from the ReCT respectively, with the dose transferred to the CBCT using rigid registration. The necessity of plan adaptation was assessed by the change in dose to 95% of the planning target volume (D95) and mean dose to the parotids.

**Results:**

The treatment plans were adapted clinically for all 18 patients but only 7 actually needed an adaptation yielding 11 unnecessary adaptations. Applying a method using the daily CBCT with the planned dose distribution would have yielded only four unnecessary adaptations and no missed adaptations: a significant improvement from that done clinically.

**Conclusion:**

Using the DIR between the planning CT and daily CBCT can flag cases for plan adaptation before every fraction while not requiring a new re‐planning CT scan and dose recalculation.

## 1 INTRODUCTION

Radiation therapy is a standard treatment option for a variety of cancers, where the precise geometric targeting of tumors can be exploited for achieving better tumor control while limiting healthy tissue damage. The specific targeting and attenuation of radiation are unique to the patient's anatomy at the time of the planning CT (PCT) simulation, but these conditions are difficult to maintain throughout an entire course of treatment due to changes in anatomy.^1^, ^2^, ^3^, ^4^ To account for changes in patient anatomy, plan modification may be required during the treatment course to ensure accurate targeting. Plan adaptation has been shown to improve treatment outcomes by promoting better tumor control and limiting toxicities,^5^, ^6^ but this procedure entails additional costs of re‐imaging, re‐planning, and additional quality assurance. Although the potential benefits of plan adaptation are obvious, no guidelines on decision‐making and optimal time for re‐planning are available.

Plan adaptation has been reported for various treatment sites including lung,^7^ prostate,^8^, ^9^, ^10^ and head and neck cancers.^11^, ^12^ Across all treatment sites, adaptation is necessary due to tumor shrinkage, weight loss or other significant anatomical changes that impact the dose distribution (e.g., lung collapse or re‐inflation). Specifically for head and neck cancers, large volume changes are common and often detected by external examination or through poor fitting of immobilization devices, but minor changes can go unnoticed. However, relatively minor anatomy changes may still have a significant effect on the dose distribution and are more difficult to discern by visual inspection of anatomy alone.

More precise and conformal radiation treatments available with modern techniques may need more plan adaptations to provide consistent target dose coverage and healthy tissue sparing with a changing anatomy. For making a decision on the necessity of plan adaptation in clinical practice, efficient daily evaluation of the delivered dose distribution on the modified anatomy is required. Different methods have been presented on detecting volume changes^13^ and landmark movements,^14^ but most rely solely on visual inspection by clinicians. These visual inspections may not be consistent as shown by inter‐observer studies.^15^ Several groups have presented adaptation strategies and schedules throughout treatment.^16^, ^17^ A recent study using the same dataset as in this study has produced a method of detecting anatomical differences to flag consideration of plan re‐evaluation without considering the dose distribution.^18^

Currently, cone beam CT (CBCT) imaging is routinely used for patient alignment and anatomy monitoring, but can also be used for dosimetric assessment of actual radiation delivery. Dose calculations on CBCTs are possible with the results varying between reported studies^19^, ^20^, ^21^ because of inferior image quality and tissue densitometry. Performing reliable analysis of the dose to the target and organs at risk would require contouring of relevant structures on the daily CBCT image. An attractive alternative is to employ deformable image registration (DIR) to transfer contour information from the planning CT study for analysis. DIR has been shown to produce a variety of results depending on the algorithm used, original contouring accuracy and imaging modalities (i.e., CT simulation, MRI or CBCT). Unfortunately, registration between different imaging modalities has been shown to have worse accuracy^22^ especially for CBCT images due to limited image quality and artifacts.

There are two primary effects of anatomical deformations on a radiation treatment: 1) movement of voxels and regions on interest (ROI) relative to the planned dose distribution and 2) change in the dose distribution itself due to re‐arrangement of voxels or density changes therein. The current gold standard (GS) for determining whether to adapt a treatment plan involves a new CT simulation (ReCT), dose calculation and DIR to map contours from the PCT. This procedure is time‐consuming and expensive but accounts for both effects of anatomical deformation and is applied when gross anatomical changes are suspected.

The best alternative without a new CT simulation involves using DIR to warp the planning CT to match the daily anatomy from the CBCT and perform dose calculation as proposed by Veiga et al.^23^ and accounts for both effects of anatomical deformations. However, the dose recalculation practically can be difficult and time‐consuming. It is usually performed off‐line which limits its routine daily use at the treatment unit. What if you could determine the necessity of plan adaptation without a new CT scan and dose calculation? Without the re‐computation of the dose, only the movement of voxels and ROI's relative to the planned dose distribution are considered, but not the change to the dose distribution. The dose distribution is assumed to be robust and only mildly affected by the re‐arrangement of the voxels. In this study, we explore the results of using the CBCT without a dose calculation and a CBCT with a dose calculation and compare both to the current gold standard. The goal is to see if assessing the movement of ROI relative to the planned dose distribution provides enough dose information to properly trigger the plan adaptation process, when compared to current clinical practice of visual inspection.

## 2 METHODS

### 2.1 Patient studies

For this study, 18 patients who received multi‐fractionated radiotherapy for head and neck cancer and had plan adaptation during treatment course were selected. Each patient had a CT scan taken before treatment (range 4–30 days) and used for planning (i.e., PCT), daily pre‐treatment CBCT studies and another CT re‐taken during treatment (ReCT) when anatomy changes were deemed significant (day “X”). Significant changes included sensitive structures moving into high‐dose regions, tumor moving out of this region or excessive weight loss by the patient. Both PCT and ReCT studies were obtained on a 120 keV Phillips Big Bore CT scanner (Philips Healthcare, Fitchburg, WI, USA) with a 512 × 512 image size, 0.9–1.2 mm resolution, and 3 mm slice thickness. CBCT scans were performed every 1–5 fractions with the on‐board imaging available on Varian iX and True Beam treatment units (Varian Medical Systems, Palo Alto, CA, USA) using 100 keV with a 512 × 512 or 384 × 384 image size, 0.5–0.65 mm resolution and 2.5–2 mm slice thickness. Treatment plans had prescribed doses ranging 50–70 Gy to the planning target volume (PTV) in 30–35 fractions using volumetric arc therapy (VMAT) with two 360° arcs and included 1 or 2 target volumes. Specifically, 15 patients had only one target, three had two targets and all patients had a larger nodal volume overlapping all targets prescribed to a lower dose. Treatment planning and dose calculations were performed on a Pinnacle treatment planning system (version 9.10, Philips Healthcare, Fitchburg, WI, USA) using Pinnacle's collapsed cone convolution superposition algorithm^24^ using a dose grid of 3 × 3 × 3 mm^3^. All image registrations (both rigid and deformable) were performed with software from MIM Maestro (version 6.5 MIM Software Inc., Cleveland, OH, USA) using the default DIR algorithm applying an intensity based free form algorithm, with a sum of squared differences similarity metric.^25^ The mean registration error using MIM Maestro between two kVCT's was shown to be 1.7 mm by Kirby et al.^22^ using a deformable Head and Neck phantom.

### 2.2 Dose distribution estimation

**Figure 1:**
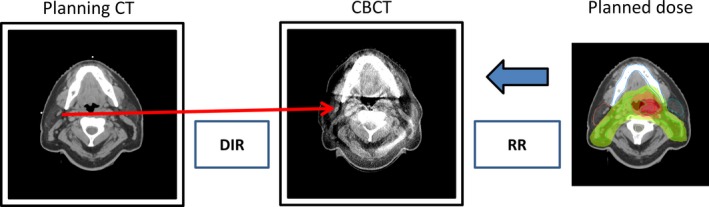
Illustration of the CBCT_P_ method for the evaluation of the need for plan adaptation using the DIR of planning CT to daily CBCT study and the planned dose distribution. DIR—deformable image registration, RR—rigid registration.

**Figure 2:**
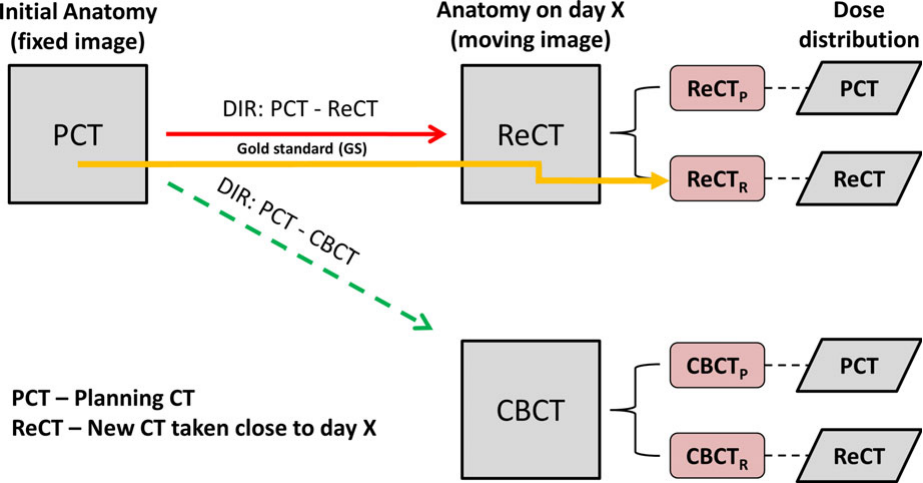
Schema describing the daily dose estimation using DIR from the planning CT to either the daily CBCT or re CT study (ReCT). Two different dose distributions computed on the PCT or ReCT are transferred to the moving image using a 6 degree of freedom rigid registration. The gold standard method is highlighted in yellow using the ReCT and recomputed dose. Day X is when ReCT was ordered due to observed significant anatomical changes.

To determine the necessity of plan adaptation, an estimation of the dose distribution “of the day” was required and three estimation methods are presented and compared to the current gold standard which requires a re‐planning CT. The first method (CBCT_P_) used DIR to map the contours from the PCT to the daily CBCT with the planned dose distribution rigidly registered to the daily CBCT as shown in Fig. 1. The second method (CBCT_R_) used the DIR to map the contours from the PCT to the daily CBCT with the recalculated dose (from the ReCT) rigidly registered to the daily CBCT. The third method (ReCT_P_) used the DIR to map the contours from planning CT to the ReCT with the planned dose distribution rigidly registered to the ReCT. The gold standard method (ReCT_R_) applied DIR to map contours from the PCT to ReCT with the recalculated dose on the ReCT. Both dose distributions (planned and recalculated) were obtained using the original treatment plan parameters and beam; the plan was not re‐optimized. The rigid registration process used 6 degrees of freedom and simulated the alignment of the CBCT study to PCT (or ReCT) performed by the radiation therapists in the clinic before each fraction. In total, four separate methods estimated the daily dose distribution using the CBCT or ReCT as the secondary CT study, with the planned or recomputed dose. For clarity, each method was referred to by the secondary image used (CBCT or ReCT) and if the planned or ReCT dose was used, denoted by subscript P or R, respectively. All dose estimation methods are illustrated in Fig. 2, showing all four investigated combinations.

### 2.3 Voxel‐to‐voxel dose comparison

The clinically relevant comparison of the dose results obtained by different estimations requires evaluation on a voxel‐to‐voxel basis. Every voxel in the PCT study can have a different dose value in fraction X (when ReCT was ordered), depending on the secondary CT study for image registration and the dose distribution. Comparison with any other method is done by calculating the relative dose difference to the GS (RD_*j*_) for a specific structure *j* across each individual voxel *i*:

(1) $${\text{RD}}_{j}=\frac{1}{18}\sum_{p}^{18}\frac{1}{N_{j}}\sum_{i}^{N_{j}}\frac{\left|D{\left(\text{GS}\right)}_{\mathrm{ip}}^{j}-D{\left(T\right)}_{\mathrm{ip}}^{j}\right|}{D{\left(\text{GS}\right)}_{\mathrm{ip}}^{j}}\times 100\%$$

between a test method (*T*) and GS averaged over all *N*_*j*_ voxels within all 18 patients *p*.

Voxel‐to‐voxel analysis was performed for the right and left parotids because they were present in all image studies, incurred significant deformation and are frequently positioned close to the target volume. The analysis was also performed for the spinal cord because it is a clinically important structure.

### 2.4 Test for the necessity of plan adaptation

In practice, the estimations of dose distribution changes would be used to determine if a current plan delivery is not within clinical dose tolerances and needs adaptation. For our dose distribution estimation methods, adaptations were considered necessary if the following dose tolerances were exceeded: mean dose to parotid equal or above to 26 Gy, max dose to spinal cord equal or above 50 Gy or dose to 95% of the PTV below the prescription dose. Using two of the methods, CBCT_P_ and CBCT_R_, dose values were calculated and compared to clinical tolerances to see which method would accurately trigger plan adaptation, when compared to the gold standard (ReCT_R_). Any parotid with a planned mean dose equal or above 26 Gy was excluded from this test since the organ was already planned to receive greater than the tolerated dose. The spinal cord is an important organ for head and neck radiotherapy planning, but was not considered for the necessity of adaptation test since the threshold for a max dose of 50 Gy was only crossed by one patient.

For the PTV, the dose was determined at each voxel using CBCT_P_ and CBCT_R_ methods. The threshold criterion for adaptation was for 95% of the volume (D95) to be below the prescribed dose. The D95 parameter was selected following recommendations for evaluating the target coverage.^26^ Only the primary PTV was analyzed for each patient.

Evaluation of the clinical decision to adapt or not relied on the compliance with both parameters: the parotid mean dose and D95 to the PTV. To simulate a conservative treatment situation the mean parotid dose and max spinal cord dose was rounded to the nearest integer, for example, 25.6 Gy is rounded to 26 Gy. The results were reported as the number of unnecessary adaptations (adapting, when within tolerance) and missed adaptations (not adapting, when tolerances were exceeded).

## 3 RESULTS

### 3.1 Voxel‐wise dose comparison

The relative dose difference RD_*j*_ given by Eq. (1) for each method are shown in Table 1 for the ipsilateral and contralateral parotids and spinal cord. The error caused by only the changed dose distribution is presented by the ReCT_P_ row and the CBCT_R_ row represents the error caused only by the DIR between different imaging modalities. CBCT_P_ row represents the error when both effects were present.

**Table 1:** Relative voxel‐wise dose difference from gold standard (ReCT_R_) (RD_*j*_) for ipsilateral and contralateral parotids and spinal cord, averaged over 18 patients. Standard deviation is displayed in brackets

Secondary image and dose distribution	Ipsilateral parotid	Contralateral parotid	Spinal cord
ReCT_P_	8% (5.7%)	7.9% (5%)	3.8% (1.6%)
CBCT_P_	12.7% (9.5%)	13.5% (7.8%)	5.7% (2.4%)
CBCT_R_	7.5% (4%)	7.7% (4.5%)	4% (2%)

### 3.2 Test for necessity of adaptation

**Figure 3:**
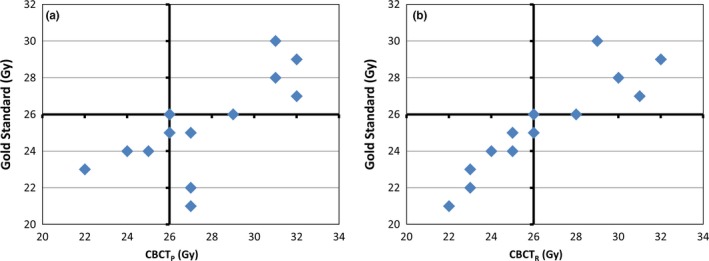
Predicted mean dose using a) CBCT_P_ and b) CBCT_R_ methods compared to ReCT_R_ (gold standard) for 15 parotid glands. Clinical threshold of 26 Gy is shown by solid lines. ReCT_R_ is the DIR to ReCT using the recalculated dose. CBCT_P_ is the DIR to daily CBCT using the planned dose. CBCT_R_ is the DIR to daily CBCT using the recalculated dose.

**Figure 4:**
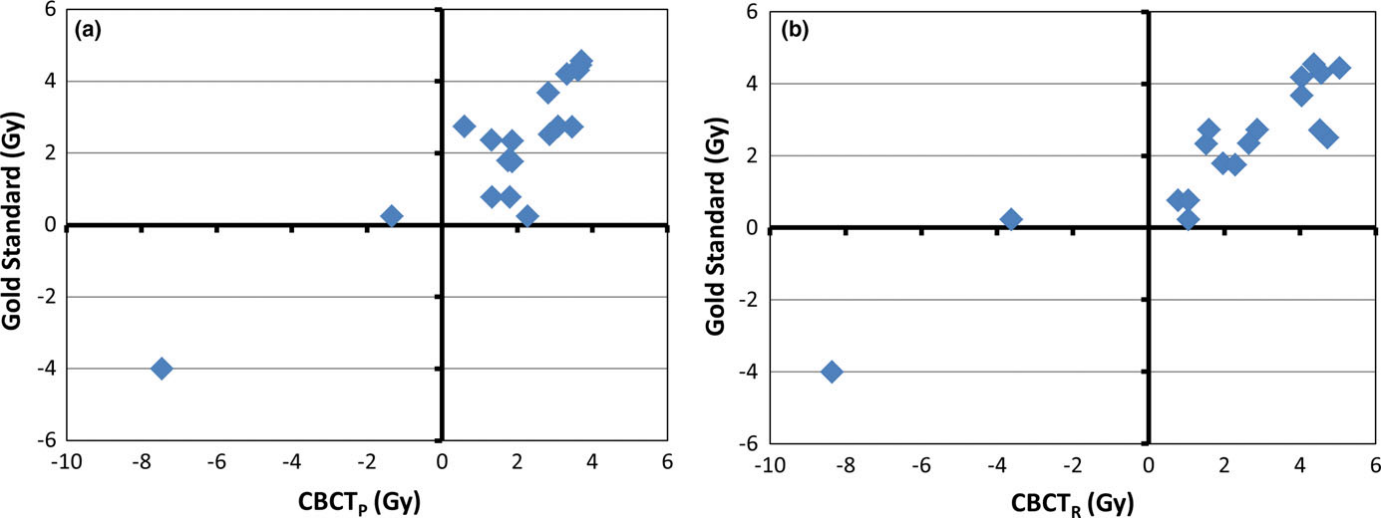
The difference between predicted D95 and the prescribed dose for the PTV for using a) CBCT_P_ and b) CBCT_R_ methods compared to ReCT_R_ (gold standard). Values are presented as the difference from the prescribed dose. Dashed line represents conservative criteria (within 1 Gy of threshold). ReCT_R_ is the DIR to ReCT using the recalculated dose. CBCT_P_ is the DIR to daily CBCT using the planned dose. CBCT_R_ is the DIR to daily CBCT using the recalculated dose.

The parotid mean dose estimates using CBCT_P_ and CBCT_R_ are compared relative to the 26 Gy threshold to the gold standard (ReCT_R_) in Figs. 3(a) and 3(b), respectively. The number of parotids that were incorrectly labelled as either greater than or less than 26 Gy, of 15 tested parotids was five for CBCT_P_ and one for CBCT_R_. The D95 estimates relative to the dose prescription for CBCT_P_ and CBCT_R_ are compared to the gold standard in Figs. 4(a) and 4(b), respectively. The number of patients where the CBCT‐based prediction was different from the gold standard on their PTV D95 parameter (of 18 patients) was one for both CBCT_P_ and CBCT_R_.

To simulate a clinical decision‐making situation, the results for both parotids and PTVs were combined to determine whether to adapt or not based on the dose predictions from CBCT_P_ or CBCT_R_ methods. Clinically, the plans for all 18 patients were adapted using a conservative approach based on anatomical changes alone but according to our dose analysis, only seven were outside of tolerance leaving 11 potentially unnecessary adaptations. Using the CBCT_P_ method there would have been only four unnecessary adaptations without missing any required adaptations. For CBCT_R_ (with the recomputed dose) there would have been two unnecessary adaptations while also not missing any required adaptations.

## 4 DISCUSSION

In a standard workflow, the only dose distribution always available is the one calculated using the initial CT simulation for planning purposes. Theoretically, the dose gradients from the planned dose distribution indicate what dose differences may occur due to specific anatomical changes. Dose gradients are mainly defined by the original beam geometry relative to the planned iso‐center, which is not affected by deformation. Without extensive deformation, these gradients can be maintained and could predict dose change, when combined with a deformation field. However, with large volume or density reductions within the beams path significant changes to the dose distribution can result, which could lead to missed adaptations, if no dose calculation is performed. But very large volume/density changes are clearly visible by visual inspection on imaging and would be flagged and trigger adaptation by a therapist.

The average relative dose differences RD_*j*_ for each organ presented in Table 1 show that for both parotids and spinal cord the RD_*j*_ from the CBCT_R_ method (which is a result of DIR error alone) is similar to the results from the ReCT_P_ method, which is the error from using the planned instead of the recomputed dose. The CBCT_P_ RD_*j*_ includes both sources of error but is less than the sum of errors in ReCT_P_ and CBCT_R_ methods.

It has been shown that DIR error is specific to the algorithm used^27^, ^28^ and image quality.^22^ In this study, only one commercial algorithm was used to evaluate the utility of applying DIR to CBCT studies using an unmodified commercial product. More accurate dose estimations could be performed if registration error was known and accounted for as demonstrated in our previous work.^29^ Typical plan adaptation strategies revolve around re‐planning on the CBCT study using DIR to propagate contours and evaluate dose.^30^, ^31^, ^32^ Two publications by Veiga et al. have evaluated the process of using DIR to CBCT studies for determining daily dose^23^ and accumulated dose with different DIR algorithms,^28^ but in both cases dose computations are needed for each fraction. In this study, DIR of the daily CBCT study is proposed to evaluate anatomical changes without a re‐scan of the patient or dose calculation using a commercial DIR algorithm.

Practically speaking, DIR procedures can help physicians to decide when to adapt their radiation treatment plans. From the results presented in Figs. 3 and 4, the clinical decision to re‐plan all 18 of these cases was not necessary, with 11 of the original patient plans still within clinical tolerances. Clinical decisions of plan adaptation were made before the re‐scan using personal experience, which explains the discrepancy in adaptation rates between our GS and that decided clinically. Our results have shown that both methods using daily CBCT studies (CBCT_R_ and CBCT_P_) yielded very conservative results and missed no required adaptations. If the simplest prediction method (CBCT_P_) was used, only four patients would have been unnecessarily re‐scanned and adapted. This demonstrates that using the DIR to the CBCT of the day without a dose calculation in CBCT_P_ method can determine when to adapt a treatment plan better than that done clinically avoiding a number of unnecessary CT simulations and re‐planning efforts. Performing an additional dose calculation in CBCT_R_ caught two additional unnecessary plan adaptations at the cost of additional computation time, while without a dose computation the procedure can be completed within one minute allowing for an efficient “adapt or not” decision online.

## 5 CONCLUSION

Improvements in IGRT and conformal radiation delivery have made adaptive radiation therapy a reality, but steps need to be taken to ensure its efficiency. Practical implementation requires an efficient method of daily evaluation and decision‐making to determine when plan adaptation is truly necessary. The method of dose evaluation using on‐board CBCT imaging alone is limited by the necessity for dose calculation, contouring and image registration. We have shown that the daily CBCT image mapped back to the planning CT without a dose calculation can provide sufficient information for the important decision of when to re‐plan. The goal is to prevent the use of unnecessary additional CT simulations and dose computations with a quick online evaluation. Further research needs to be performed with more patients and other treatment sites including abdomen and thorax and for treatment techniques that will produce a different landscape of dose gradients.

## CONFLICT OF INTEREST

The authors declare no conflict of interest.
